# Food Compass and the challenge of sustainability on the route towards healthful diets

**DOI:** 10.1038/s41598-024-57615-9

**Published:** 2024-03-22

**Authors:** Luca Muzzioli, Francesco Frigerio, Matteo Mazziotta, Lorenzo Maria Donini, Alessandro Pinto, Eleonora Poggiogalle

**Affiliations:** 1 Department of Experimental Medicine, Sapienza University, 00185 Rome, Italy; 2 Italian National Institute of Statistics, 00184 Rome, Italy

**Keywords:** Healthy diet, Sustainable diet, Climate change, Nutrient profiling system, Front-of-pack-labels, FOPL, Food Compass score, FCS, Agribalyse database, Health policy, Nutrition

## Abstract

In order to tackle the global increase in overweight and obesity prevalence, several nutrient profiling systems have been developed; among others, Food Compass Score (FCS) has been designed to encompass multiple domains of food healthfulness. However, environmental sustainability of healthy diets is another crucial dimension which should not be overlooked in the context of human health. The aim of the present study is to assess the association between healthiness and environmental sustainability of food items, using the FCS and Agribalyse databases, respectively. A total of 806 matching food items were identified, grouped in 12 food categories; within each category, differences in median Z-scores between FCS and Single Environmental Footprint (EF) Score were assessed. While Fruits, Legumes and Nuts, Mixed foods, Meat Poultry and Eggs (MPE), Savory and Sweets, and Vegetables showed statistically significant differences (all p < 0.001), Beverages (p = 0.361), Dairy (p = 0.092), Fats and Oils (p = 0.594), Grains (p = 0.436), Sauce and Condiments (p = 0.093), and Seafood (p = 0.241) had similar Food Compass and Single EF Z-scores distributions. These findings underscore a relevant lack of difference between healthfulness and environmental impact of some prominent food categories, such as Grains and Seafood. Therefore, we suggest matching nutrient profiling systems with adequate environmental sustainability indices.

## Introduction

Nutrient profiling systems (NPSs) are an area of growing interest within public health research. They build on the premise that ranking foods according to their nutritional value can help consumers make informed choices at the Point-of-Purchase^1^. Notwithstanding, their ability to positively influence eating behaviors is still under scrutiny, especially when being used in Front-of-Pack labeling^2^. Their algorithms usually include few key nutrients, such as Total Fats, Saturated Fats, Sugars, Sodium, Proteins, Fibers, and food energy content^3^. In this scenario, the development of Food Compass Score (FCS) by Mozaffarian et al.^4^ was the first attempt to include not only a considerable number of macro- and micro-nutrients, but also other non-nutritional factors pertaining to a healthful diet. The presence of the NOVA classification system^5^ among the FCS attributes represents an interesting innovation, since emerging research has pointed at the frequent consumption of ultra-processed foods (UPFs) and processed foods (PFs) as a modifiable risk factor for Non-Communicable Diseases (NCDs) incidence^6^. Recently, O’Hearn et al.^7^ validated Food Compass by observing that a diet enriched with high FCS food items was positively correlated with a reduced incidence of NCDs; furthermore, the study by Detopoulou et al.^8^ found a positive correlation with the level of adherence to Mediterranean diet. According to recent observations from our group^9^, there is an urgent need to consider both healthfulness and environmental sustainability of diets, in view of the fact that current climate change, pollution and resource depletion can be mitigated by scaling down global food demand^10^. Moreover, dietary patterns can steer food consumption towards low emission foods and thus become an effective mitigation option to the increasing trend of global warming^11^. Developing a system which conveys information on nutritional values, level of industrial processing and environmental footprint of foods might orient consumers towards adequate and sustainable dietary patterns. In this regard, the group of Clark et al.^12^ assessed the healthfulness and environmental footprint of 57,000 foods, using the Nutri-score and a novel summary environmental index, respectively. A previous work from our group displayed a weak-to-moderate correlation between the included NPSs and indicators of environmental sustainability^13^, whereas healthy and sustainable diets (e.g., the Mediterranean diet and the diet developed by Willet et al.^11^) proved to be strongly correlated. Moreover, NPSs based on portions showed a higher degree of correlation than NPSs developed on a 100-g reference scale. Therefore, the aims of the present study were to build a larger dataset combining nutritional healthfulness with environmental sustainability indicators and to examine whether and to which extent FCS may be associated with the environmental footprint of food items.

## Methods

### Food Compass score

Food Compass score (FCS) is a recent NPS encompassing 54 attributes (e.g., the unsaturated:saturated fat ratio, the fibre:carbohydrate ratio, the presence of additives, the level of food processing) grouped into 9 domains deemed relevant to food healthfulness (i.e., nutrient ratios, vitamins, minerals, food ingredients, additives, processing, specific lipids, dietary fiber & protein, and phytochemicals). Assigning different weights to specific attributes and domains, the overall score is computed by adding up the domain scores, which are based on their respective attributes. The final FCS is adjusted across all food categories so that items can take up a minimum score of 1 (least healthy) and a maximum of 100 (healthiest). According to the creators, the frequency of consumption of a specific food item should be minimized when FCS ≤ 30, moderated when 31 ≤ FCS ≤ 69 or promoted when FCS ≥ 70. The FCS was firstly applied to 8032 foods included in the 2015–2016 What We Eat In America (WWEIA) NHANES survey, based on 24-h dietary recalls.

### Agribalyse database

Agribalyse is a French research project launched in 2010 with the purpose of producing public Life Cycle Inventories of agricultural products^14^. Since the beginning the database has progressively included 2520 food items, assessing their environmental impacts through the use of Life Cycle Assessments. The perimeter considered in each item assessment is from cradle to grave which includes data on all steps of the food product life cycle such as farming, processing, packaging, transportation, retail and consumption. Currently, it provides values of 16 major environmental indicators as well as a composite index, the Single Environmental Footprint (EF) score that is measured in mPt/kg of product. The higher the value of the Single EF score, the higher the environmental impact. At the time of the study, the last available database version was Agribalyse 3.0 (ADEME source, AGRIBALYSE data v3.0—2020).

### Statistical analysis

Statistical analysis was carried out with SPSS (IBM SPSS Statistics for Windows, Version 27.0. Armonk, NY: IBM Corp.). As a first step, we relied on the open-access French database Agribalyse 3.0 and the Food Compass Database (FC-DB). 8032 and 2520 food items were identified in the FC-DB and the Agribalyse database, respectively; correspondence between each item was assessed, leading to the final inclusion of 806 food products (Agribalyse-matched database, AM-DB). The Shapiro–Wilk test was performed, suggesting a non-normal distribution of FCS across all food categories; therefore, the non-parametric Mann–Whitney test was applied to assess differences in Food Compass Score (FCS) between the original FC-DB and AM-DB, across all food categories. Due to different measurement units, FCS and Single EF score were transformed into standard scores (Z-scores) before comparing their respective values^15^. The Wilcoxon Signed-Rank Test was used to determine differences between Food Compass and Single EF Z-score distributions for each food category. Finally, single plots and boxplots of FCS and Single EF scores were used to graphically display original values and standard scores. Outliers and extreme values were identified using Tukey’s method^16^ and displayed in boxplots as empty circles and asterisks, respectively.

## Results

In order to analyze the relationship between environmental sustainability and healthfulness of the FCS, we matched the Food Compass database (FC-DB) with the Agribalyse 3.0 database; the resulting database (Agribalyse-matched database, AM-DB) included n = 806 food items out of 8032 (original FC-DB). The AM-DB can be found in the Supplementary material.

The absolute and relative number of items across food categories in the AM-DB are displayed in Table 1.

**Table 1:** Absolute and relative frequency of items across food categories in AM-DB and FC-DB.

Food category	AM-DB (n)	% total	FC-DB (n)	% total
Beverages	40	4.96	275	3.42
Dairy	58	7.20	245	3.05
Fats and oils	14	1.74	129	1.61
Fruits	58	7.20	264	3.29
Grains	54	6.70	727	9.05
Legumes, nuts and seeds	48	5.96	264	3.29
Mixed dishes	78	9.68	2206	27.47
Meat, poultry and eggs	94	11.66	763	9.50
Sauces and condiments	27	3.35	160	1.99
Savory, sweets and desserts	132	16.38	1000	12.45
Seafood	75	9.31	434	5.40
Vegetables	128	15.88	1565	19.48
Total	806	100.00	8032	100.00

*AM-DB* Agribalyse-matched Database, *FC-DB* Food Compass Database.

**Figure 1:**
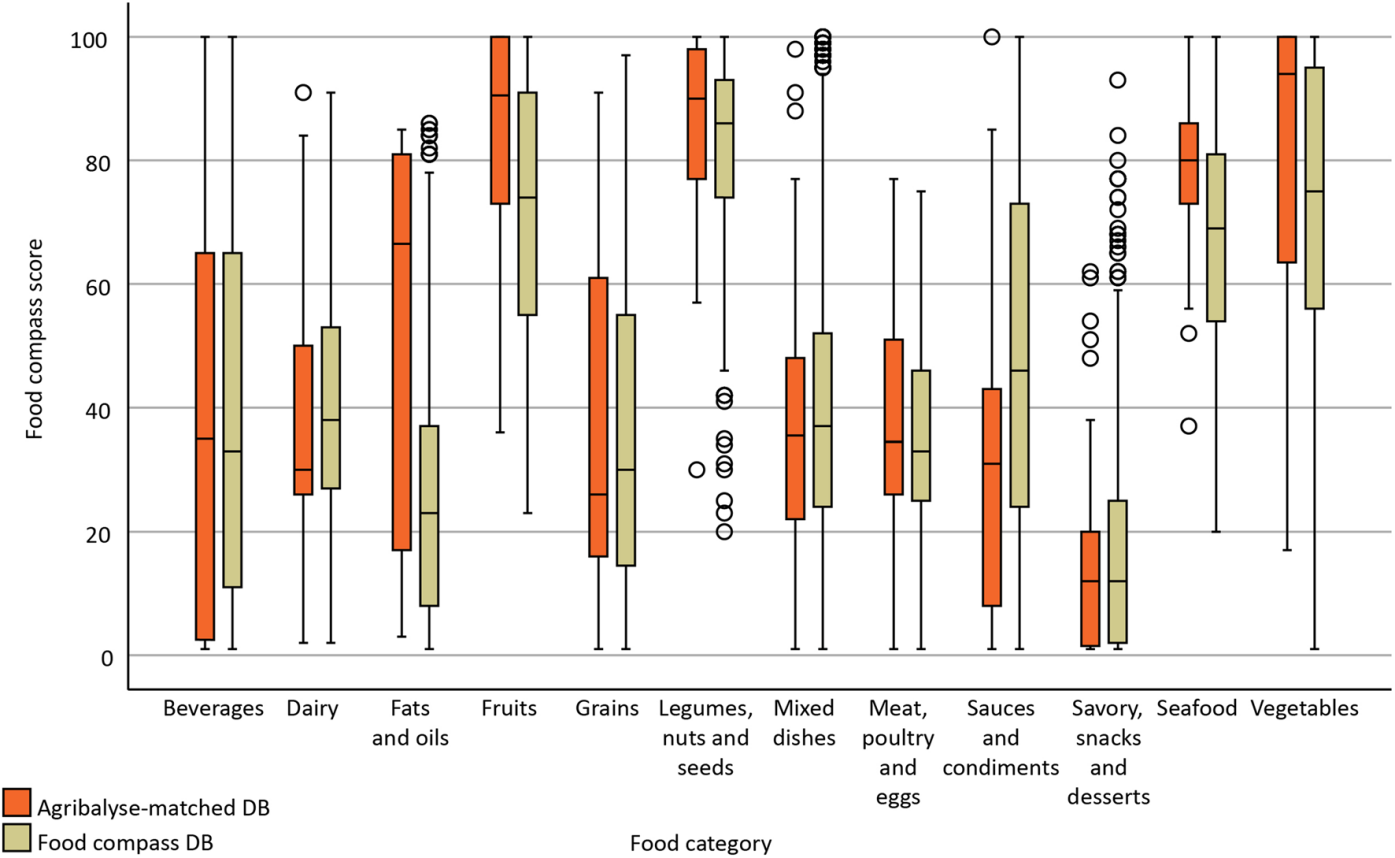
Paired boxplot of Food Compass Scores of the Food Compass and the Agribalyse-matched databases, grouped by food category. Outliers are depicted by empty circles, while extreme values by asterisks. *DB* database.

Figure 1 displays the variability of FCS across different food categories both in the Agribalyse-matched DB (AM-DB) and the original Food Compass DB (FC-DB). The following groups were found to have different distributions between the two databases: Fats and oils (p = 0.010), Fruits (p < 0.001), Sauces and condiments (p = 0.004), Seafood (p < 0.001), and Vegetables (p < 0.001); no statistically significant differences were found across the remaining food categories (all p > 0.1).

**Figure 2:**
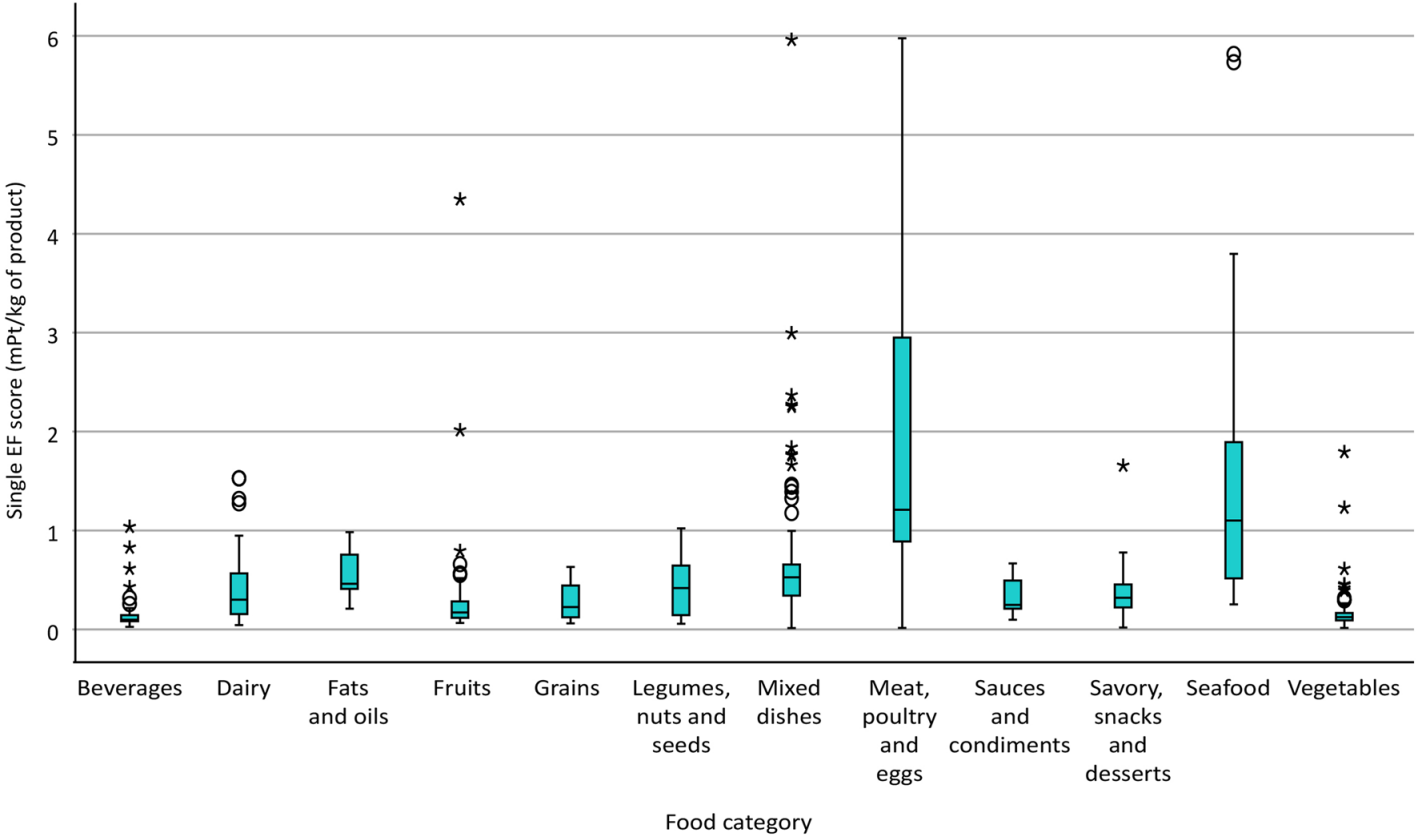
Boxplots of the single EF score across food categories in the Agribalyse-matched database. Outliers are depicted by empty circles, while extreme values by asterisks. *EF* Environmental Footprint.

Figure 2 depicts the graphical distribution of single EF scores across all food categories in the AM-DB. The highest median scores were observed for Meat, poultry, and eggs (MPE) and Seafood, whereas Beverages, Fruits, and Vegetables scored the lowest. A similar pattern was seen for EF score variability, where Seafood and MPE displayed the broadest variability while, on the contrary, Beverages, Fruits, and Vegetables showed the lowest variability.

**Figure 3:**
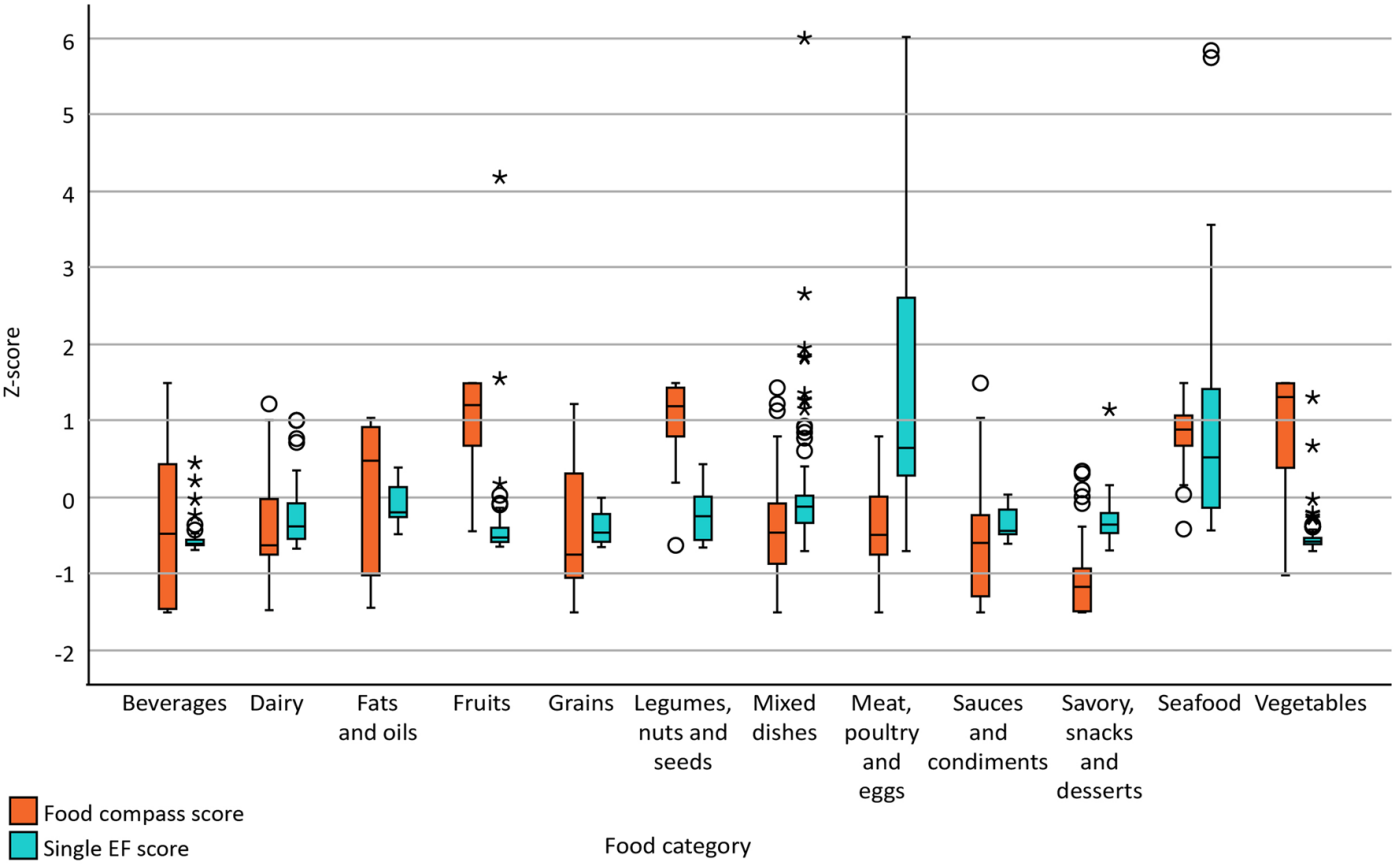
Paired boxplots of Food Compass (orange) and single EF Z-scores (light blue) grouped by food category. *EF* Environmental Footprint.

**Figure 4:**
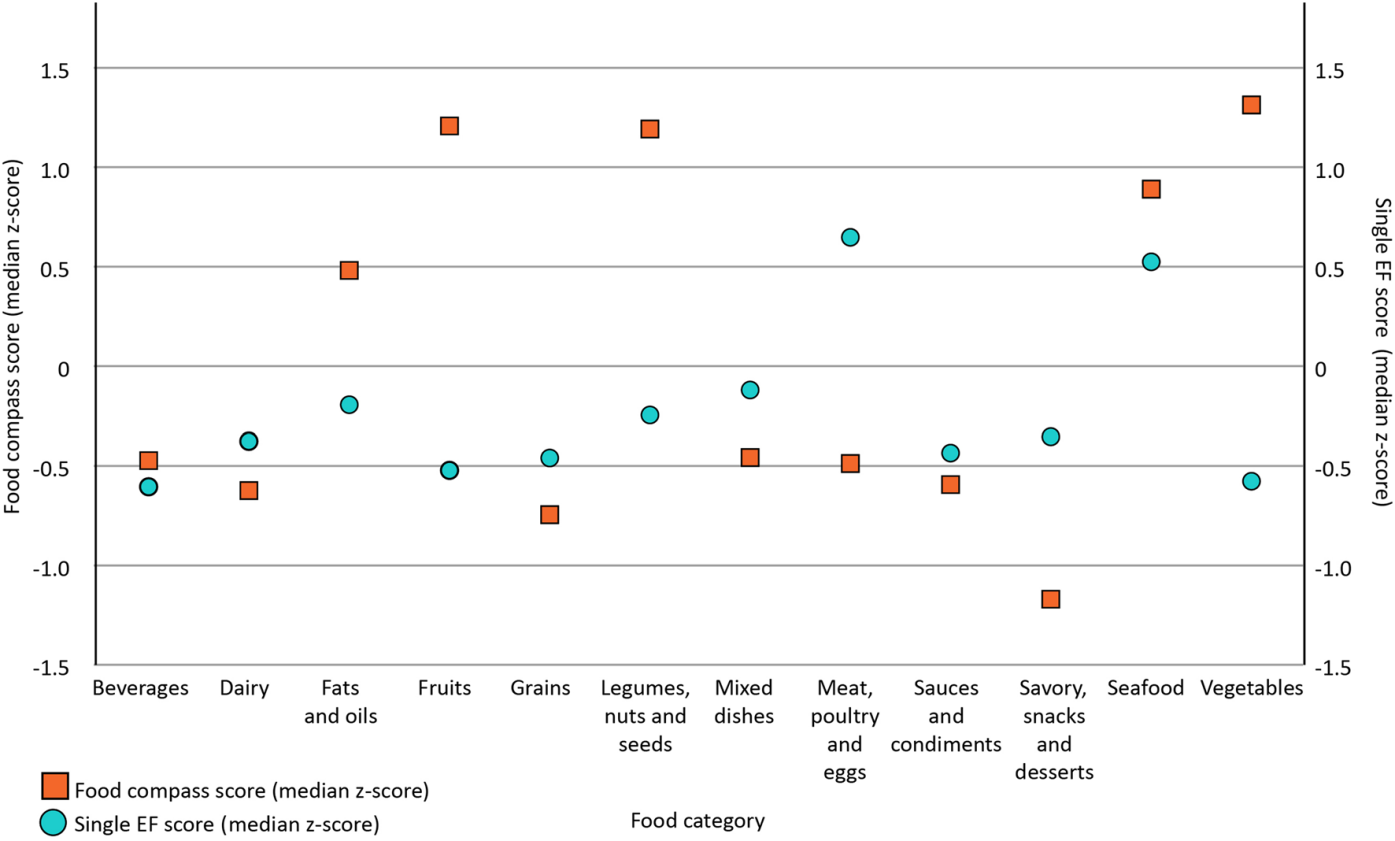
Plot of the Food Compass (orange squares) and single EF median Z-scores (light blue circles) across food categories. *EF* Environmental Footprint.

Figure 3 shows the paired boxplots of the Food Compass and single EF Z-scores across food categories. Beverages (p = 0.361), Dairy (p = 0.092), Fats and oils (p = 0.594), Grains (p = 0.436), Sauces and condiments (p = 0.093), and Seafood (p = 0.241) had similar Food Compass and single EF Z-scores distributions, while Fruits (p < 0.001), Legumes, nuts and seeds (p < 0.001), Mixed dishes (p < 0.001), MPE (p < 0.001), Savory, sweets and desserts (p < 0.001) and Vegetables (p < 0.001) showed statistically significant differences. Visual inspection of median standard scores appears coherent with these observations (Fig. 4) where environmental impact and Food Compass scores are depicted by light blue circles and orange squares, respectively. Food categories with reduced environmental impact (i.e., lower EF median Z-scores), ranked high in healthfulness (i.e., higher Food Compass median Z-scores): this is the case of Fruits, Legumes, nuts and seeds, and Vegetables. Conversely, food categories with greater environmental impact (i.e., higher single EF median Z-scores) scored low in healthfulness (i.e., low Food Compass median Z-scores): examples are the Mixed dishes and MPE categories. Differently, Grains scored relatively low both in Food Compass and single EF median Z-scores while Seafood scored relatively high in both median Z-scores.

## Discussion

The present study delves into the potential relationship between food environmental sustainability and healthfulness scales. We previously showed^13^ the rank correlation between different NPSs and a tailored environmental composite index^17^; however, the FCS was not included at the time and the dataset was limited in terms of number of food items (n = 182). Therefore, matching the Agribalyse 3.0 DB with the FC-DB led us to expand the number of food items (n = 806), food categories and environmental indicators. The Agribalyse 3.0^14^ provides reference data on the environmental impact of agricultural and food products through a database built according to the life cycle analysis (LCA) methodology^18^.

Similar to the FCS, which is a summary health scale resulting from fifty-four attributes, the single EF score is a composite index of the sixteen environmental indicators of food items^19^. As for single EF scores, the marked variability (i.e. wider boxplots) of MPE and the limited variability of Fruits, Vegetables, and Grains were both expected a priori: MPE includes a wide spectrum of animal products, while the above-mentioned plant-based food groups are inherently homogenous and have consistently displayed a reduced environmental impact across studies. Furthermore, a significant difference between their respective standard FCS and single EF scores is coherent with international guidelines favoring plant-based foods (to be consumed on a daily basis and sufficiently sustainable) against MPE (to be consumed sparingly and highly pollutant)^20,21^. Nonetheless, the relatively low median Food Compass Z-scores of the Grains category, on top of the lack of difference between its FCS and single EF Z-scores, is not in line with the scientific principles of healthy and sustainable dietary patterns, such as plant-based and flexitarian diets where grains are the main staple in terms of carbohydrate and energy content^22^. Low FCS for Grain products could lead to an increased consumption of less nutritious and less sustainable food groups. Likewise, the high Food Compass Z-scores for the Seafood category and the non-significant difference between Food Compass and single EF median Z-scores may induce a more frequent consumption of this specific food group, with a worse dietary pattern from both a nutritional and an environmental standpoint. Nonetheless, the objective of the study was not to propose a synthesis of healthiness and environmental sustainability messages into a single score but rather to verify the congruence between the two domains so that the results of this analysis may help the developers of Food Compass and other NPSs define their strategy on how to tackle food sustainability.

The novel need for a parallel and integrated assessment of both the nutritional and environmental domains in the context of dietary patterns was already captured by Clark et al.^12^ who proposed a composite environmental score (i.e., the Environmental Impact Score) and paired it with a Nutriscore-derived nutritional index (i.e., Nutrition Impact Score). When comparing nutritionally adequate food items to their respective environmental impact, quite surprisingly a discrepancy emerged, paving the way to potentially misleading information for consumers^1^. These heterogeneous and divergent findings may be related to the choice of a nutritional index relying on a 100 g-scale, i.e. the Nutriscore. Our findings somewhat concur with observations reported by Clark et al.; in our case, the analysis was conducted on the FCS which was not specifically designed for packaged food and, thus, can be applied to different settings of food preparation (e.g., house, restaurants).

We acknowledge some limitations to our study. Data on food LCA were retrieved from the Agribalyse 3.0, which is the most comprehensive European database, whereas the FCS was originally computed on the U.S. 2015–2016 Food and Nutrient Database for Dietary Studies (FNDDS). Nonetheless, no U.S. public food environmental footprint database was available at the time of the present research and, on the other hand, no bromatological data were available within the Agribalyse 3.0 DB, which would have let us compute FCS autonomously. Another limit is that, after the matching procedure, only a sample of the original food items (806 out of 8032) were included in the AM-DB. Nevertheless, the relative representation of each food category was comparable with the FC-DB for seven out of twelve categories, while the most penalized category (i.e. Mixed) includes mostly UPFs and PFs, which are not reflective of a healthful and sustainable dietary pattern^23,24^. Furthermore, to the best of our knowledge, this is the first study to investigate the association between FCS and food environmental impact.

Accordingly, FCS shows a positive correlation not only with other NPSs and dietary patterns (i.e. HSR, Mediterranean Diet Score^13^), but also with low-grade inflammation and cardiovascular disease markers^25^. On the other hand, recent studies have shown that it may be positively biased towards PFs and UPFs enriched with phytochemicals, penalizing minimally processed or unprocessed food ingredients; this may be partly ascribed to unbalanced weighing factors for the included attributes^26,27^. Furthermore, the overall score is computed on 100 kcal of the selected food item: this choice appears more reasonable than 100 gr-based NPSs since it facilitates “the use of a single scoring algorithm for a diverse range of items, from a single small item to a food with mixed ingredients or a large mixed dish or meal, even among items that differ greatly in bulk. Scoring per 100 kcal was also considered valuable for scaling up to compare diverse combinations that may be sold and consumed together—for example, to score an entire shopping basket, an entire diet or an entire portfolio of foods being sold by a particular vendor”^4^. As suggested by our previous work, where NPSs using the per-portion reference displayed a slightly better association with environmental impact indicators^13^, further research should investigate 100 g, 100 kcal or serving (portion) sizes according to the national and international dietary guidelines^28^.

In agreement with recent studies^11^ and the last Intergovernmental Panel on Climate Change report^10^, including environmental sustainability is pivotal to improve individual dietary patterns. To date, no existing NPS incorporates both nutritional and environmental information, and the challenge of how to encompass sustainability in food healthiness communication has yet to be resolved. Consequently, in spite of the preliminary nature of our data, we suggest that present and novel NPSs would consider such a crucial dimension. Despite emerging data suggesting the association of some NPSs with all-cause mortality and non-communicable diseases^29^ further research should be prompted to define an effective integrated measure not only in terms of sustainability but also for primary and secondary prevention strategies and policies. Finally, on the back of the objective issues faced in combining food healthfulness and environmental footprint, it would be advisable to start building comprehensive databases.

### Supplementary Information

Supplementary Information.

## Data Availability

The dataset used in the present work (AM-DB) is the result of a matching process between the Agribalyse 3.0 DB (https://simapro.com/products/agribalyse-agricultural-database/) and the Food Compass DB (https://static-content.springer.com/esm/art%3A10.1038%2Fs43016-021-00381-y/MediaObjects/43016_2021_381_MOESM1_ESM.pdf). These DB are property of their respective owners. The food items dataset included in this research paper can be found in the Supplementary material section.
